# Supplementary material for: Intermittent Fasting Reduces Neuroinflammation and Cognitive Impairment in High-Fat Diet-Fed Mice by Downregulating Lipocalin-2 and Galectin-3
Source: Nutrients. 2024 Jan 3;16(1):159. doi: 10.3390/nu16010159 (PMC10780385; doi:10.3390/nu16010159)
Supplement: Supplementary file 1 [file nutrients-16-00159-s001.zip › nutrients-2735809-supplementary.pdf]

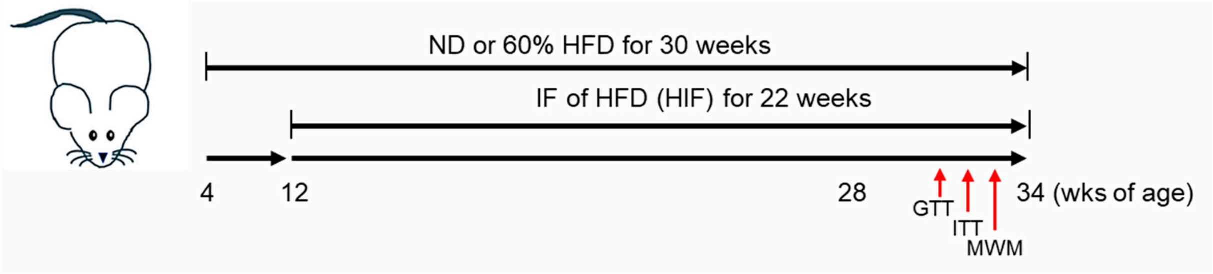

Supplementary Figure S1. Experimental scheme of IF protocol in HFD mice. Mice in the ND and HFD groups were fed a ND or HFD for 32 weeks, whereas mice in the HIF group were fed an HFD for 8 weeks and then switched to an IF protocol consisting of alternating 24-h periods of fasting and feeding for 22 weeks. ND; normal diet, HFD; high-fat diet, HIF; HFD + intermittent fasting (IF), GTT; glucose tolerance test, ITT; insulin tolerance test, MWM; Morris water maze.

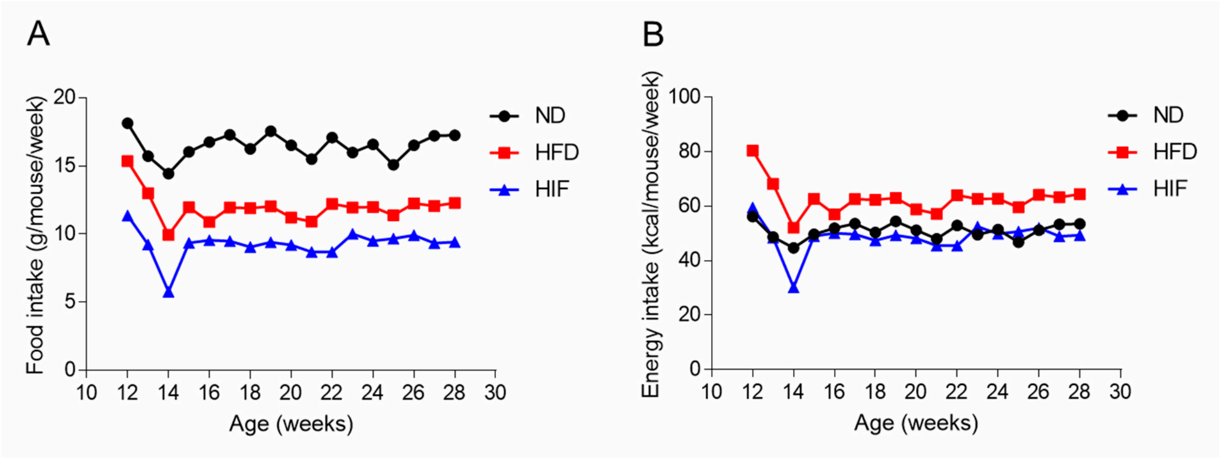

Supplementary Figure S2. Effects of IF on food intake and energy intake in HFD mice. (A) Food intake and (B) Energy intake every other day for 16 weeks at 12 weeks.

Supplementary Table S1. List of primary antibodies

Antibody	Company	Catalog No.	Dilution(s)	Applications	Source
F4/80	Santa Cruz	sc-377009	1:200	IF	Mouse
Perilipin-1	Abcam	ab61682	1:200	IF	Goat
Bax	Santa Cruz	sc-7480	1:1000	WB	Mouse
Bcl-2	Santa Cruz	sc-492	1:1000	WB	Rabbit
LCN2	R&D	AF1857	1:1000,<br>1:200	WB<br>IF	Goat
GAL3	Santa Cruz	sc-23938	1:1000<br>1:200	WB<br>IF	Rat
α-tubulin	Sigma	T5168	1:5000	WB	Mouse
MMP9	Abcam	ab38898	1:1000	WB	Rabbit
ICAM-1	BD Bioscience	BD554967	1:1000	WB	Mouse
ZO-1	Santa Cruz	sc-33725	1:1000	WB	Rat
Claudin-5	Thermo Fisher	35-2500	1:1000	WB	Mouse
AQP4	Santa Cruz	sc-9888	1:200	IF	Goat
Albumin	Abcam	ab192603	1:200	IF	Rabbit
TNF-α	Santa Cruz	sc-1351	1:1000	WB	Goat
TNFR1	Santa Cruz	sc-8436	1:1000	WB	Mouse
IL-6	MyBioScience	mbs3007753	1:1000	WB	Rabbit
HMGB1	Abcam	ab18256	1:1000	WB	Rabbit
RAGE	Abcam	ab3611	1:1000	WB	Rabbit
GFAP	Sigma	G3893	1:1000	IF	Goat
Iba-1	Wako	019-19741	1:200	IF	Rabbit
β-actin	Sigma	A5441	1:3000	WB	Mouse

Abbreviations: AQP4, aquaporin4; GAL3, galectin-3; GFAP, glial fibrillary acidic protein; HMGB1, high mobility group box1; ICAM-1, intercellular adhesion molecule-1; IF, immunofluorescence; IL-6, interleukin-6; LCN2, lipocalin-2; MMP9, matrix metalloproteinase 9; RAGE, receptor for advanced glycation end products; TNF- α, tumor necrosis factor-α; TNFR1, tumor necrosis factor receptor1; WB, western blot; ZO-1, zonula occludens-1.
